# Lithotomy—Semilunar External Incision

**Published:** 1869-03

**Authors:** James T. Newman

**Affiliations:** Chicago, Illinois


ARTICLE XIII.

### LITHOTOMY—SEMILUNAR EXTERNAL INCISION.

BY JAMES T. NEWMAN, M.D., Chicago, Illinois.

Was consulted by G. H., September 9th, 1868, who stated to me that he had great difficulty in passing his urine. Oftentimes, when in the act of micturition, the stream would flow freely; then, all at once, it would stop suddenly; at other times it would start, and he could not control it. There was great pain in passing the fæces; there was also a constant dull pain at the neck of the bladder, together with a sense of weight, or pressure, at the lower part of the pelvis. His urine was high-colored, and often mixed with blood. He begged of me, for God's sake, to give him something to relieve him; at the same time, asked me what was the matter? I was not prepared to give him an answer so soon—putting all these symptoms together, told him nothing more than that there was an urinary disease of some kind, of which, I could not determine at the present time; well knowing that they might be considered diagnostic of enlargement of the prostate, or irritation of the neck of the bladder. Sometimes, where there is a tumor hanging by a small pedicle, it will give rise to symptoms similar to those mentioned.

We will now take those disorders up, and examine them, *seriatim.* An enlarged prostate gland is attended with symptoms similar to those of a stone; with this difference, that the motion of a carriage does not increase the pain when the prostate is diseased; while, in a case of stone, the patient suffers the most excruciating pain. .t also generally happens that the fits of stone come on at intervals, whereas the pain from an affected prostate is neither so unequal nor acute. When there is inflammation of the neck of the bladder, the parts become tumefied; hence the obstruction to the urinary jet, which gives rise to the conditions so often mistaken for those of stone.

These thoughts that I have here tabulated flew through my mind quicker than it takes to write them. I, however, told him that I considered his case to be one of a very grave nature, but would give him all the relief in my power, but would have to subject him to an examination, or, in other words, sound him. He very readily consented. I placed him in proper position, and took a small beaked sound and commenced exploring the urethra and bladder: he fainted, but I threw some water in his face, which soon revived him, and continued my exploration. It was not long before the sound struck upon a hard body, and gave forth the well-known click. I now was no longer in doubt about what was the matter, and advised an operation, to which he at once consented. I told him I had never performed the operation. He expressed confidence in me, and that I might just as well commence on him as any other person. I must confess that the man startled me with the coolness he manifested, and, at the same time, my vanity was somewhat flattered; being anxious to record my name amongst surgeons that have performed this brilliant operation. I resolved to undertake it; but, after having made every preparation myself, confidence was taken down when I remembered that some poet had said, that "fools rush in, and stand where angels fear to tread;" but, thus far I was committed, and there was no backing out. Dr. W. H. Horn kindly promised to assist me; and, on the 20th of September, 1868, at 10 o'clock in the morning, we commenced to operate. The patient was properly secured, and put under the influence of chloroform. I passed a rectangular grooved staff into the bladder: the stone was immediately felt. I proceeded to make a transverse, crescentic incision in the perineum, after the manner of Erichsen: the centre of the incision was half an inch above the anus, and each extremity of it about one-half inch from the tuber ischü. I cut down, in the line of the superficial incision, to the central part of the perineum, in order that I might separate the bulbous portions from the rectum. I now directed the knife into the groove of the staff, through the membranous urethra, and just in front of the prostate. Its blade being directed upwards, the knife was now drawn backwards, cutting upwards, so as to form a vertical incision in the superficial surface, about one inch in length in the middle line, commencing with the original crescentic incision. Having laid aside the knife, a lithotomy caché was introduced along the groove of the staff; and, by the withdrawal of this, both lateral lobes of the prostate were divided to the extent of about three-fourths of an inch. The lithotomy forceps were introduced; and, by dexterous manipulation, the stone was seized and withdrawn. The calculus was phosphatic; in form, an oblate spheroid; its transverse diameter, 3.75 inches; its conjugate, 1.45 inches. He bore the shock well; expressed himself greatly relieved; recovered rapidly; and, on the 10th of October, the urine ceased to flow through the wound, and passed through the natural passage: it soon afterwards healed up, and, at this time, he is in perfect health.

